# Regulator of Chromosome Condensation 2 Modulates Cell Cycle Progression, Tumorigenesis, and Therapeutic Resistance

**DOI:** 10.3389/fmolb.2020.620973

**Published:** 2021-01-13

**Authors:** Kun Guo, Cheng Zhao, Bin Lang, Huiqin Wang, Hang Zheng, Feng Zhang

**Affiliations:** College of Life Sciences, Shanghai Normal University, Shanghai, China

**Keywords:** regulator of chromosome condensation 2, chromosomal passenger complex, tumorgenesis, cancer therapeutic resistance, DNA damage

## Abstract

Accurate regulation of cell cycle is important for normal tissue development and homeostasis. RCC2 (Regulator of Chromosome Condensation 2) play a role as chromosomal passenger complex (CPC) implicated in all cell cycle phases. RCC2 was initially identified as Ran guanine exchange factor (GEF) for small G proteins. Therefore, RCC2 plays a key role in oncogenesis of most cancers. RCC2 is implicated in Colorectal Cancer (CRC), Lung Adenocarcinoma (LUAD), breast cancer, and ovarian cancer. Expression level of RCC2 protein determines regulation of tumor cell proliferation, invasion, metastasis, and radio-chemotherapeutic resistance. In this review, we explored proteins that interact with RCC2 to modulate tumor development and cancer therapeutic resistance by regulation of cell cycle process through various signaling pathways.

## Introduction

Regulator of Chromosome Condensation 2 (RCC2) is a member of Regulator of Chromatin Condensation 1 (RCC1) superfamily. Genes of this family comprise one or more RCC1-like domains (RLDs) involved in protein-protein interactions (Hadjebi et al., 2008). Proteins in this superfamily play significant roles in nucleocytoplasmic transport (Riddick and Macara, 2005), ubiquitinoylation (Scheffner and Staub, 2007), cell cycle, and response to DNA damage (Tan and Lee, 2004; Choi et al., 2013; Smith et al., 2014). RCC2, also known as Telophase Disc-60 (TD-60), was initially identified as a telophase disk-binding protein during mitosis, as it mediates progression of prometaphase to metaphase (Andreassen et al., 1991; Mollinari et al., 2003). RCC2 gene has a mitotic phosphorylation motif implicated in regulation of protein localization during mitosis (Yang et al., 2007). Furthermore, previous studies report that RCC2 gene plays an essential role in regulating cell cycle progression during interphase (Yenjerla et al., 2013). In addition, RCC2 acts as a specific Guanine Exchange Factor (GEF) for both Rac1 and RalA proteins (Mollinari et al., 2003). Notably, RCC2/RalA are implicated in regulation of kinetochore-microtubule interactions during prometaphase stage of mitosis (Papini et al., 2015). Moreover, Hu et al. report that RCC2 plays a pivotal role in G2-M transition (Hu et al., 2018).

The function of RCC2 in tumor development has been studied extensively in recent years due to its involvement in tumor cell migration. For example, RCC2 interacts with Fibronectin-dependent (FN-dependent) adhesion signaling pathways, by inhibiting activation of Rac1 and Arf6, which regulate adhesion complexes, thereby facilitating continuous cell migration (Danen, 2009; Humphries et al., 2009). A previous study shows that RCC2 overexpression promotes metastasis of Lung Adenocarcinoma (LUAD) by inducing Epithelial-Mesenchymal Transition (EMT) through modulation of mitogen-activated protein kinase-c-Jun N-terminal kinase (MAPK -JNK) signaling pathway (Pang et al., 2017). MAPK-JNK is a potential autophagy regulation pathway positively correlated with lung cancer tumorigenesis (Shih et al., 2012; Wang et al., 2014; Zhou et al., 2015). Additionally, RCC2 interacts with and deactivates Rac1, which is controlled by p53 (a short-lived tumor suppressor protein) signaling axis. These interactions regulate cell migration and suppression of metastasis in colorectal cancer (Song et al., 2018). Moreover, RCC2 plays an oncogenic role in breast cancer, by activating Wnt-signaling pathway, thus promoting cell proliferation and migration through Epithelial-mesenchymal transition (EMT) (Chen et al., 2019). Further, RCC2 is implicated in tumor cell proliferation (Matsuo et al., 2013; Chen et al., 2019; Yu et al., 2019), apoptosis and sensitivity (Wu et al., 2018; Gong et al., 2019; Yu et al., 2019), and poor prognosis of microsatellite stable (MSS) tumors (Bruun et al., 2015). Although several studies on RCC2 have been conducted, no review has previously reported its functions. Therefore, this review explores the functions of RCC2 in cell cycle and roles of RCC2 in cancer progression.

In this review, we present important aspects of RCC2 biology. The review summarizes the role of RCC2 in cell cycle, effects of overexpression of RCC2 gene in development of various cancers, such as promotion of tumor cell metastasis, RCC2 molecular features and proteins that interact with RCC2 as a tumor-promoting gene.

## Gene and Protein Structures

**Figure 1:**
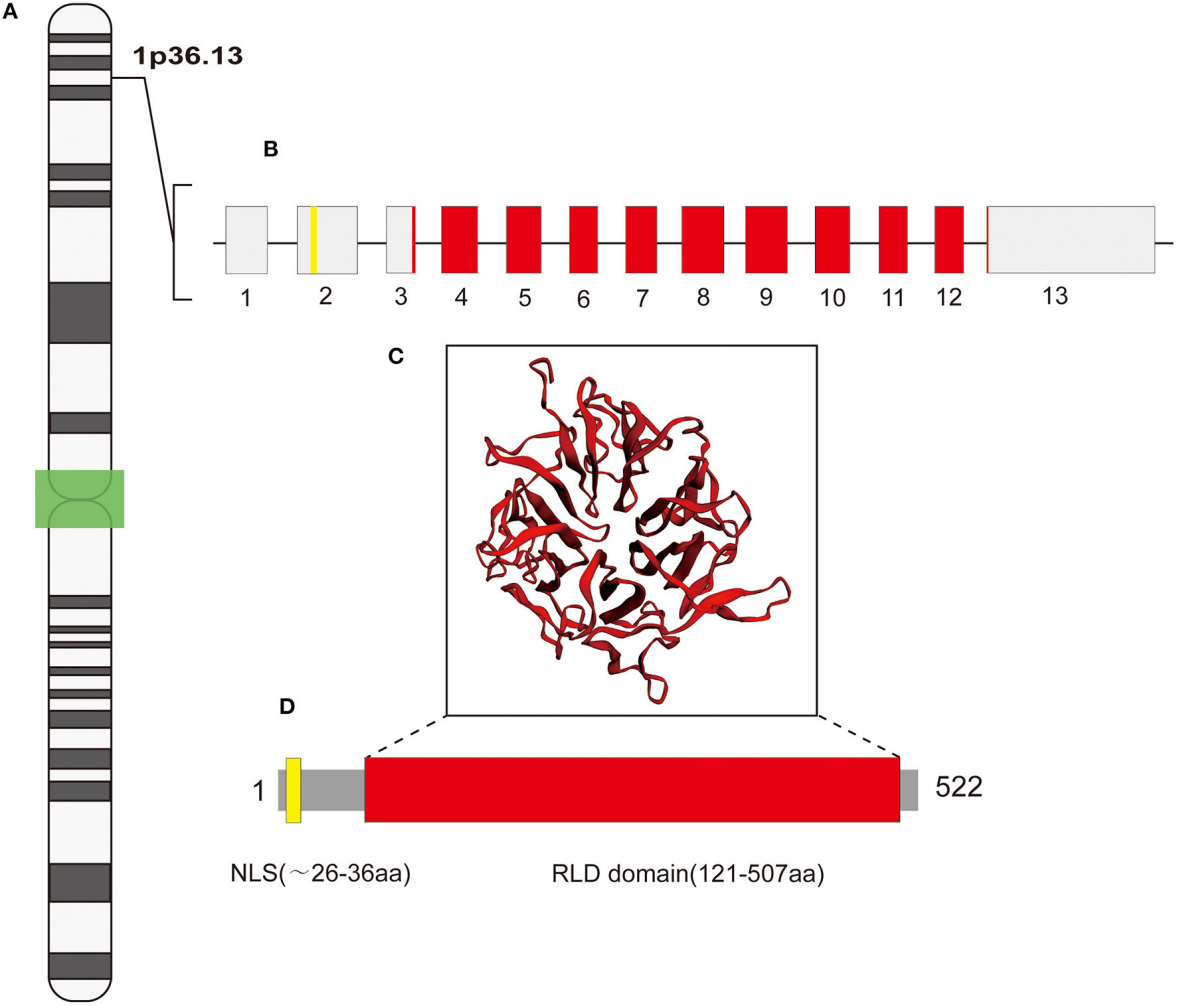
A representation of RCC2 gene and its products. **(A)** The gene is located on chromosome 1p36.13; the structure on the left shows the chromosome cytobands, with the centromere (shown in green). **(B)** The gene is composed of 13 exons, whereby exon 2 encodes the NLS motif (depicted in yellow); RLD domain is encoded by the 3′ end of exon 3 to the 5′ start of exon 13 (in red). **(C)** RLD domain (in red) stretching from the middle to the carboxyl-terminal region. The inset represents β -propeller secondary structure of RLD domain [predicted by Phyre2 (Kelley et al., 2015)]. **(D)** The protein structure comprises 522 amino acid residues that includes an NLS motif at its amino-terminal region, starting approximately at the 26th residue and ending at the 36th residue (yellow box).

Regulator of Chromosome Condensation 2 (RCC2) (OMIM accession # 609587) is encoded by a 4040 bp gene composed of 13 exons and located on the short arm of chromosome 1 (1p36.13) (Figure 1). RCC2 expression occurs during the late G2 phase of the cell cycle (Mollinari et al., 2003). Despite its location in the nucleus, RCC2 plays different roles in the cytoskeleton, plasma membrane, centromere, chromosome, and midbody (Andreassen et al., 1991; Martineau-Thuillier et al., 1998; Mollinari et al., 2003; Yenjerla et al., 2013; Williamson et al., 2014). A previous study reports that p53 binds to a palindromic motif in the promoter region of RCC2 to regulate its transcription (Song et al., 2018). Notably, RCC2 gene encodes a 522-amino acid protein which contains a conserved nuclear localization signal (NLS) in its amino-terminal region (Figure 1). A nuclear localization signal is an amino acid sequence that “tags” a protein for translocation into the nucleus by nuclear transport. RCC2 protein accumulates in the inner centromere region of chromosomes during prophase and redistributes to the midzone of the mitotic spindle during anaphase (Andreassen et al., 1991; Martineau-Thuillier et al., 1998; Mollinari et al., 2003). RCC2 protein structure consists of a RCC1-like domain (RLD) near the carboxyl-terminal region (Figure 1) (Ohtsubo et al., 1987). The structure of RLD comprises a seven-bladed propeller, which plays a role in protein-protein interaction, and in binding DNA (Ohtsubo et al., 1987; Aebi et al., 1990; Seki et al., 1996; Renault et al., 1998). RLD in RCC2 comprises five repetitive elements formed by 51–75 amino acid residues (Figure 1). Interestingly, Song et al. reported that the RLD domain of RCC2 binds to Rac1 through a β-hairpin comprising seven-bladed propeller structure thus inactivating it (Song et al., 2018).

## RCC2 and Cell Cycle

Cell cycle is a complex process, comprising a series of events that strictly control cellular growth and division. Several proteins directly or indirectly interact with the continuously changing chromatin during cell cycle to ensure that chromosomes are accurately segregated into two daughter cells. Therefore, cell cycle is essential for normal growth and development of organisms.

**Figure 2:**
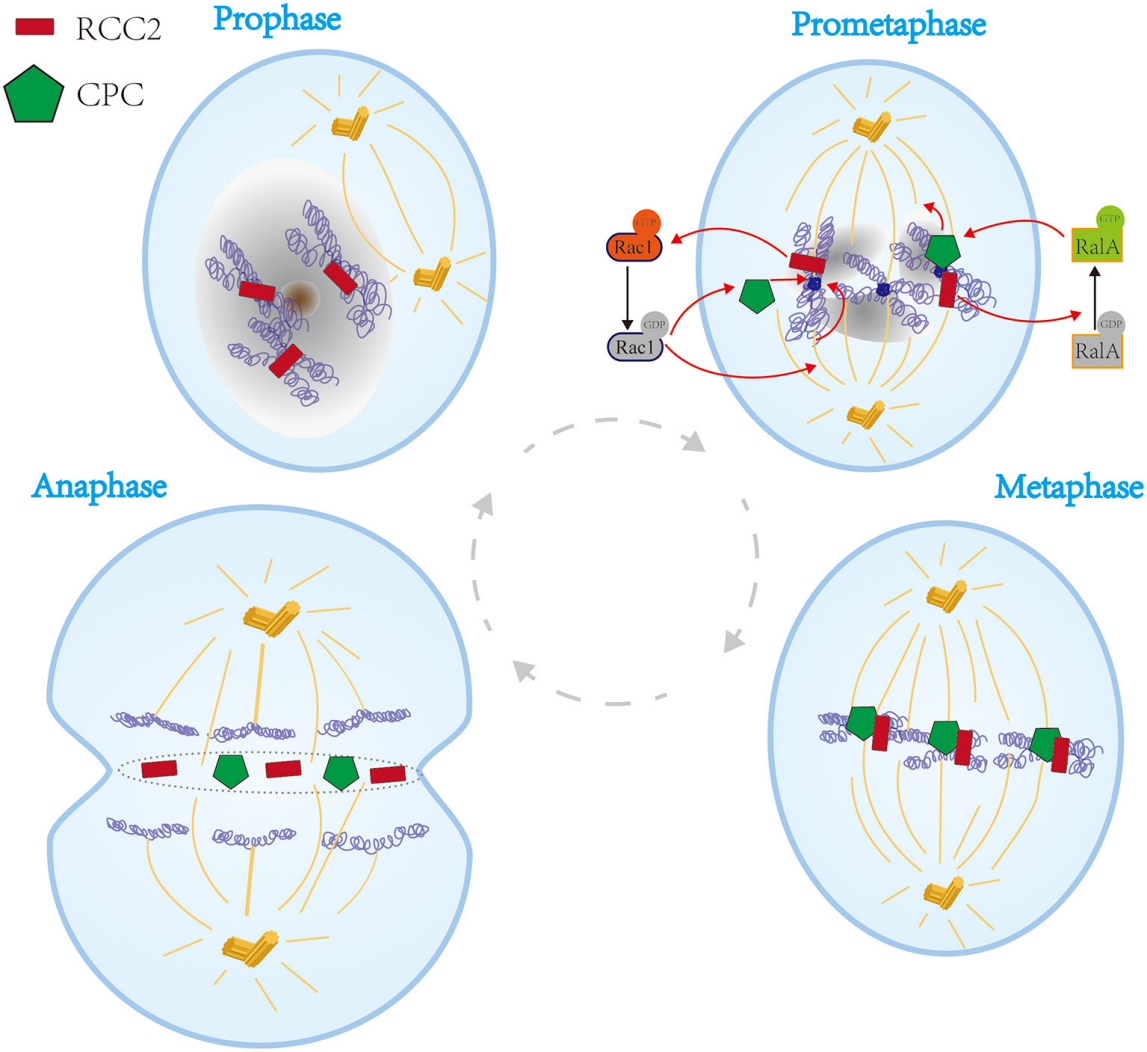
Schematic representation of the functions of RCC2 in the cell cycle process. **In prophase**, RCC2 is located at the chromosome primary constriction. **In prometaphase**, RCC2 binds to microtubules and converts GTP-Rac1 to GDP-Rac1, which plays a vital role in association of kinetochores with spindle microtubules and association of passenger proteins with inner centromeres. Moreover, RCC2 acts as a GEF for RalA, which converts GDP-RalA to GTP-RalA thus modulating kinetochore-microtubule interactions by regulating CPC. **In metaphase**, RCC2 migrates to the equator together with CPC. **In anaphase**, RCC2 is incorporated into the telophase disc organelle, thus fully partitioning the cell at the spindle equator in late anaphase and telophase.

In 1991, Paul et al. reported a new mammalian mitotic organelle, which they named “Telophase Disc-60xl0^3^Mr” (TD-60) which was later confirmed as RCC2 by HUGO Gene Nomenclature Committee (HGNC) (Andreassen et al., 1991). The protein was identified in human autoimmune serum that revealed its role in cytokinesis (Andreassen et al., 1991). During prophase, RCC2 is located at the primary constriction of the chromosome. RCC2 dissociates from chromosomes until mid-anaphase, thus aligning with microtubules in the region between the sister chromatids (Andreassen et al., 1991). Thereafter, RCC2 migrates to the equator and becomes part of the microtubule-independent organelle (Andreassen et al., 1991). Subsequently, RCC2 is incorporated into a complex together with motor protein. The complex migrates to the plus end of the interpolar microtubules in anaphase and is finally incorporated into the telophase disc organelle. The complex then fully partitions the cell at the spindle equator in late anaphase and through telophase (Figure 2) (Andreassen et al., 1991).

Previous studies report that RCC2 is a member of passenger protein family, also known as chromosomal passenger complex (CPC). With the complex comprises Aurora B kinase (Adams et al., 2000), INCENP (Cooke et al., 1987), and Survivin (Gassmann et al., 2004). CPC regulates chromosomal alignment, spindle assembly, and cell cleavage during mitosis (Figure 2) (Mollinari et al., 2003). However, Diana et al. reported that RCC2 is a component of the complex but only associates with and functions as a CPC (Mollinari et al., 2003; Papini et al., 2015). Notably, RCC2 functions are vital for accurate completion of cytokinesis. Moreover, binding of RCC2 to microtubules and nucleotide-free form of the small G protein Rac1 is essential for interaction of kinetochores with spindle microtubules and passenger proteins at inner centromeres (Mollinari et al., 2003). Studies using G2/M arrested in Rac1 mutants showed that binding of RCC2 to microtubules regulates transition from prometaphase to metaphase and G2/M progression (Figure 2) (Mollinari et al., 2003; Bruun et al., 2015). In addition, RCC2 converts GTP-Rac1 to GDP-Rac1 (Figure 2) (Song et al., 2018). A new mitotic phosphorylation motif reported in RCC2 regulates protein localization during mitotic progression, thus regulating cell cycle process (Yang et al., 2007). Furthermore, RCC2 modulates activation of Rac1 and acts as a GEF for RalA. Therefore, RCC2/RalA modulates kinetochore-microtubule interactions by regulating CPC in prometaphase during mitosis (Figure 2) (Papini et al., 2015). Therefore, RCC2 plays an essential role during transition from prometaphase to metaphase and G2/M, whereas RCC1 is only implicated in G1 phase (Dasso et al., 1992; Moore, 2001).

Previous studies report that RCC2 interacts with Rac1 and Arf6 cell signaling, the interphase cell cycle progression related component (α5β1) integrins, and cortactin, implying that RCC2 plays a role in interphase (Humphries et al., 2009; Grigera et al., 2012). Mythili et al. report that loss of RCC2 affects normal cell cycle progression of G2 and suppresses G1/S (Yenjerla et al., 2013). These findings imply that RCC2 plays an essential role in regulating cell cycle progression during interphase.

In summary, low expression levels of RCC2 inhibits the cell cycle, through G1 and G2 arrest and blocking of prometaphase, thus inhibiting cell proliferation. Therefore, RCC2 plays a significant role in the cell cycle process, especially during mitosis and cell division.

## Role of RCC2 in Cancers

As mentioned earlier, RCC2 protein plays an important role in mitosis (Mollinari et al., 2003). Proteins involved in mitosis are highly expressed in various tumor cells implying that they play a role in cancer progression. Notably, RCC2 is highly expressed in several cancer types, including breast, ovarian, lymphoma, cervical, breast, gastric, colorectal, lung, and liver cancer (Chen et al., 2019).

### RCC2 and Colorectal Cancer

Colorectal Cancer (CRC) is the fourth highest cause of cancer-related deaths worldwide (Brody, 2015). High incidence rate of CRC can be attributed to adoption of western diets and lifestyles in most parts of the world (Brody, 2015). Several genomic analyses show that RCC2 is one of the commonly mutated genes in CRC (Cancer Genome Atlas Network, 2012; Giannakis et al., 2014). RCC2 is a highly conserved protein implicated in prognosis of colorectal cancers, including microsatellite instable (MSI) tumors and microsatellite stable (MSS) tumors (Kim et al., 2002). However, studies report that RCC2 is implicated in colorectal carcinomas through MSI induced by DNA mismatch repair (MMR) deficiency (Kim et al., 2002).

Furthermore, protein expression levels of RCC2 are positively correlated with development of CRC. The (A)10 mononucleotide repeat located in the 5′UTR of RCC2 and 5′UTR region also implictaed in translational regulation (Pickering and Willis, 2005). Deletion of a single base in the (A) 10 repeat decreases RCC2 expression, whereas RCC2 knockdown causes MSI tumor arrest at the G2-M phase and increased levels of apoptosis (Bruun et al., 2015). Consequently, RCC2 has plays an oncogenic role in MSI tumors. However, low protein expression level of RCC2 is associated with poor prognosis of MSS, which is attributed to its functional inhibition of tumor cell metastasis by regulating integrin α5β1-fibronectin (FN) signaling pathway (Humphries et al., 2009; Bruun et al., 2015). A study by Gautam and colleagues showed that RCC2 is a novel p53 transcriptional target that interacts with small GTPase Rac1 to inhibit its activation.

Interestingly, depletion of RCC2 in human colon cancer cell line (HCT116) showed elongated cellular morphology and increased cell migration; however, cell proliferation differences were not observed (Song et al., 2018). Activation of Rac1 in cell migration has been reported in previous studies (Pankov et al., 2005; Frank et al., 2006). In colorectal cancer, RCC2 interacts and deactivates Rac1, which is controlled by p53 signaling axis. Deactivation of Rac1 regulates cell migration and suppresses metastasis in colorectal cancer. In conclusion, mechanism of RCC2 in CRC is complex, therefore, further studies should be carried out to explore the molecular mechanism of RCC2 in development of CRC.

### RCC2 and Lung Adenocarcinoma (LUAD)

Lung cancer is among the leading cause of cancer deaths, contributing about one-quarter of global cancer-related mortalities (Siegel et al., 2020). Adenocarcinoma is the most common histologic type of lung cancer worldwide (Cancer Genome Atlas Research Network, 2014). RCC2 is a poor independent prognostic factor for LUAD patients. RNA-seq analysis of data retrieved from The Cancer Genome Atlas (TCGA) database data showed overexpression of RCC2 in LUADs (Pang et al., 2017). High expression level of RCC2 is positively correlated with T status of the tumor, lymph node metastasis, advanced clinical stage, and poor overall survival in LUAD patients (Pang et al., 2017).

In LUAD, RCC2 is associated with EMT and extracellular matrix remodeling, which contributes to tumor metastasis (Pang et al., 2017). RCC2-transfected cells show high expression levels of JNK1/2 (Pang et al., 2017). In addition, LUAD samples show high RCC2 expression, which is positively correlated with high JNK1/2 activation (Pang et al., 2017). These findings show that RCC2 plays a role in JNK pathway which is implicated in progression and maintenance of phenotypic and cellular changes associated with EMT (Sahu et al., 2015). Furthermore, JNK inhibitor inhibits RCC2-induced EMT and expression of matrix metalloproteinases (MMPs) (MMP-2 and MMP-9). The-Jun N-terminal kinases (JNKs) which are members of mitogen-activated protein kinase (MAPK) family mediate important physiological processes (Zeke et al., 2016). JNK pathway, one of the major signaling cassettes of MAPK signaling pathway, plays an important role in apoptosis, inflammation, cytokine production, and metabolism (Zeke et al., 2016). EMT is a key event during metastasis and plays a critical role in tumor invasion and metastasis during tumor progression by regulating epithelial markers, mesenchymal markers, and transcription factors (Thiery et al., 2009; Valastyan and Weinberg, 2011). In this case, activation of phosphorylated JNK is mediated by expression level of RCC2 in human LUAD cell. Moreover, activation of JNK inhibits RCC2 activity in LUAD cell, including enhanced cell motility and invasiveness, RCC2-induced EMT and expression of MMP-2 and MMP-9. On the other hand, RCC2 overexpression promotes LUAD cell proliferation and promotes metastasis by inducing epithelial-mesenchymal transition (EMT) by modulating MAPK-JNK signaling pathway (Pang et al., 2017).

In addition, previous studies report that long non-coding RNAs (lncRNAs), ENST00000439577, are associated with expression of RCC2 and promotion of proliferation, invasion, and migration of non-small cell lung cancer (NSCLC) (Feng et al., 2016).

### RCC2 and Breast Cancer

Breast cancer is the most common type of cancer in women worldwide (DeSantis et al., 2017; Siegel et al., 2020). A recent study reports that RCC2 is significantly highly expressed in breast cancer and is associated with poor overall survival in breast cancer patients (Chen et al., 2019). Previous studies report that RCC2 is highly expressed in basal-like subtype of breast cancer which is associated with higher propensity for metastasis and worse prognosis compared to other types of breast cancers (Carey et al., 2010; Chen et al., 2019). A recent study shows that RCC2 expression promotes estrogen receptor-positive (ER+) breast tumorigenesis by increasing expression of IGF1 and TWIST1, tumor-enhancing genes, and IL-6 (Wang W. et al., 2020).

Additionally, overexpression of RCC2 promotes cell proliferation and migratory capability in MCF7, and MDA-MB-468 human breast cancer cells. Therefore, RCC2 knockdown inhibits tumor progression and metastatic potential *in vivo* and *in vitro* (Chen et al., 2019). Subsequently, increased RCC2 expression promotes mesenchymal morphology and acquired migratory capability. Furthermore, overexpression of RCC2 promotes EMT progression, whereas RCC2 knockdown inhibits EMT (Chen et al., 2019). In addition, high expression levels of RCC2 regulates Wnt signaling genes, such as β-catenin, Cyclin D1 and c-Myc (Chen et al., 2019). Wnt signaling pathways are a group of signal transduction pathways implicated in physiological initiation and progression of breast cancer (Chu et al., 2004). A previous study reports that repression of Wnt/β-catenin signaling can prevent EMT, which further inhibits metastasis of basal-like breast cancer (DiMeo et al., 2009). Expression and nuclear translocation of β-catenin increases with increase in RCC2 expression levels (Chen et al., 2019). Moreover, RCC2 expression increases activation of β-catenin transcriptional targets including c-Myc and CyclinD1, further promoting progression of EMT, and resulting in breast cancer metastasis. In summary, RCC2 promotes development of breast cancer by inducing EMT and regulating Wnt-signaling pathway.

In conclusion, RCC2 functions as an oncogene in breast cancer.

### RCC2 and Ovarian Cancer

Ovarian cancer is a common gynecologic malignancy in women that leads to gynecological-cancer-associated death (Holmes, 2015). Shipeng et al. report that RCC2 is implicated in the progression of ovarian cancer (Gong et al., 2019). Expression level of RCC2 is significantly higher in DDP-resistant ovarian cancer cells compared with DDP-sensitive ones. These finding implies that RCC2 plays a vital role in drug resistance (Gong et al., 2019). RCC2-RalA signaling pathway promotes ovarian cancer cell proliferation, migration, and inhibits apoptosis (Gong et al., 2019). In addition, RCC2 interacts with RalA and regulates RalA signaling pathway, resulting in cisplatin-resistance in ovarian cancer (Gong et al., 2019). RCC2 gene is a novel target for miR-331-3p that negatively regulates it (Buranjiang et al., 2019). Decreased expression of miR-331-3p promotes growth of ovarian cancer cells by reducing expression levels of RCC2 (Buranjiang et al., 2019). However, increased RCC2 expression levels in ovarian cancer restores capacity of cell proliferation, migration, and invasion inhibited by miR-331-3p (Buranjiang et al., 2019). Therefore, RCC2 and associated proteins are potential therapeutic targets for treatment of ovarian cancer.

### RCC2 and Other Cancers

MiR-29c tumor-suppressor in gastric carcinoma targets the 3′ untranslated region (3′UTR) of RCC2, reducing its expression, thus regulating tumor cell proliferation (Matsuo et al., 2013). A previous study reports that RCC2 promotes cell growth and motility by regulating the level of RalA-GTP and modulating MAPK/JNK pathway (Wang P. et al., 2020). Furthermore, RCC2 plays a role in stabilization and transcriptional activation of Sox2, an important transcription factor that promotes malignancy of esophageal cancer (Calderon-Aparicio et al., 2020). In glioblastoma, RCC2 is implicated in tumor proliferation, tumorigenicity, and promoting radio-resistance by activating DNA methyltransferase 1 (DNMT1) transcription in a p-STAT3 dependent manner (Yu et al., 2019). Two independent genome-wide SNP association analyses showed that RCC2 plays a role in tumorigenesis (Stacey et al., 2008). In addition, RCC2 is among the candidate genes which are associated with single nucleotide polymorphism (SNP) rs7538876, which is associated with cutaneous basal cell carcinoma (BCC) (Stacey et al., 2008). Therefore, RCC2 plays a role in early recurrence of melanoma (Rendleman et al., 2013), and hepatocellular carcinoma (Xiong et al., 2019). Moreover, a recent study reported that RCC2 promotes development of hepatocellular carcinoma (HCC), especially during tumor invasion, and is implicated in cisplatin resistance (Chen et al., 2020).

## RCC2 and Therapeutic Resistance

**Figure 3:**
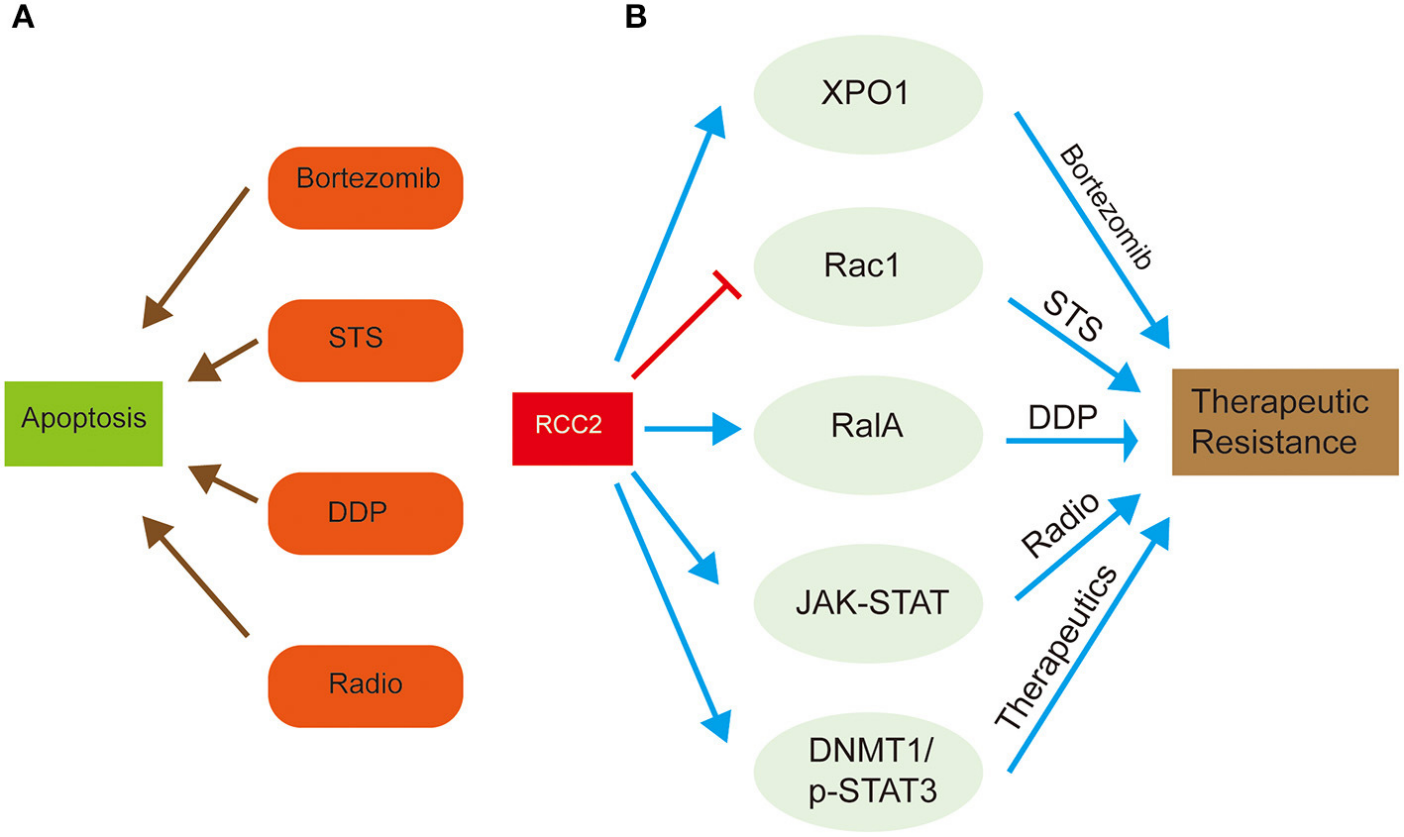
Schematic representation of the role of RCC2 in therapeutic resistance of cancer treatment through regulation of related proteins. **(A)** The left side shows the common chemotherapy drugs, bortezomib, STS, and DDP, and radiotherapy, which are used for cancer treatment by inducing tumor cell apoptosis. **(B)** The right side shows how RCC2 regulates some special proteins, (Xpo1, Rac1, RalA), and signaling pathways (JAK-STAT, DNMT1/p-STAT3), which result in attenuated anti-cancer effect of therapeutics, leading to therapeutic resistance.

Extensive studies provide in-depth understanding of the functions of RCC2 in development of various cancers, and its association with therapeutic resistance. High expression level of RCC2 is reported in lung and ovarian cancers, where it functions as a GEF for Rac1, which is subsequently involved in superoxide-induced cell death (Williamson et al., 2014; Wu et al., 2018). Rac1 functions as a proapoptotic regulator that responds to various types of apoptotic stimuli and modulates anti-proapoptotic events (Wu et al., 2018). Forced RCC2 expression in tumor cells attenuates sensitivity of tumor cells to spontaneous or Staurosporine (STS)-induced apoptosis (Wu et al., 2018). However, activation of Rac1 inhibits RCC2-induced apoptosis. Therefore, overexpression of RCC2 in tumor cells prevents cellular apoptosis and promotes chemotherapeutic resistance by blocking Rac1 signaling (Figure 3) (Wu et al., 2018). Bioinformatic studies show that RCC2 is associated with Exportin-1 (XPO1), which is a crucial factor in a complex mechanism of bortezomib resistance in multiple myeloma (Figure 3) (Chanukuppa et al., 2019). This chemoresistance results in high mortality and incurable hematological malignancy.

Cisplatin (DDP) is the first-line drug for treatment of ovarian cancer. However, drug resistance lowers therapeutic outcomes in ovarian cancer patients (Gong et al., 2019). A previous study reports that expression level of RCC2, RalA, and RalBP1 is significantly higher in DDP-resistant ovarian cancer cell lines compared to the DDP-sensitive tissues (Gong et al., 2019). These finding implies that RalA and RalBP1 are involved in oncogenesis and chemoresistance of lung cancer (Drake et al., 2007; Kashatus, 2013; Gong et al., 2019). RCC2 play an oncogenic role by promoting proliferation and migration of DDP-resistant ovarian cancer cell lines and inhibiting cell apoptosis by regulating RalA signaling pathway (Gong et al., 2019). The interaction between RCC2 and RalA is implicated in chemoresistance of ovarian cancer. Furthermore, RalA knockdown activates DDP-resistant ovarian cancer apoptosis, which is inhibited by overexpression of RCC2 (Figure 3) (Gong et al., 2019). These findings imply that RCC2 regulates RalA signaling pathway by interacting with RalA, thus promoting cisplatin resistance in ovarian cancer.

In another study on glioblastoma (GBM), expression of RCC2, the activity of the cell cycle, mismatch repair, and JAK-STAT pathways were significantly increased, implying that RCC2 plays a role in radioresistance (Yu et al., 2019). Previous studies report that RCC2 silencing decreases radioresistance of GBM cells, demonstrating the fundamental role of RCC2 gene in therapeutic resistance (Yu et al., 2019). Moreover, -STAT3 regulates transcription of DNA methyltransferase 1 (DNMT1) (Yu et al., 2019). DNMT1 and p-STAT3 (downstream factors of RCC2) are implicated in therapeutic resistance in GBM tumor cells by functioning in an epistatic manner with RCC2 (Yu et al., 2019). Therefore, RCC2 plays a vital role in tumor proliferation, tumorigenicity, and promotes radioresistance by activating transcription of DNA methyltransferase 1 (DNMT1) through a p-STAT3 dependent pathway (Figure 3).

Taken together, RCC2 promotes therapeutic resistance in most cases via interacting with various signaling pathways, which resulted in poor effects in cancer treatment. Therefore, RCC2 could be considered to be a potential biomarker. Considering the expression level of RCC2 in the development of a cancer treatment plan may be a useful method. Likewise, taking measures to inhibit the high expression of RCC2 in some cancers also may bring a good therapeutic effect.

## RCC2 and Tumor Cell Metastasis

Metastasis is a major cause of most cancer-related deaths and is a major clinical challenge in cancer treatment (Santiago-Medina and Yang, 2016). Previous studies report two forms of cell migration in tumors (Santiago-Medina and Yang, 2016). Chemotaxis is an extensively studied migration process where random cell migration, invasion, and metastasis are driven by a series of signals generated through soluble cues (Santiago-Medina and Yang, 2016). On the other hand, haptotaxis, a rarely studied mode of migration, is a substrate-bound cue-mediated mode of cell migration where the gradient of the extracellular matrix (ECM) is sensed as a guidance cue for directional migration of tumor cells (Santiago-Medina and Yang, 2016).

In addition to RCC2 being involved in multiple steps of pro-tumorigenic phenomena, it is associated with tumor metastases. As mentioned earlier, RCC2 and Rac1 signaling pathways play a vital role in therapeutic resistance (Wu et al., 2018). In colorectal cancer, RCC2 plays a vital role in the regulation of tumor migration and metastasis through p53 (a tumor-suppressor gene)-RCC2 signaling (Muller et al., 2011). p53 plays numerous roles in tumor development, it binds to a palindromic motif in the promoter region of RCC2, thus activating its transcription (Song et al., 2018). Moreover, a previous study reports that RCC2 plays a vital role in regulating directional cell movement through fibronectin-activated signaling pathways (Byron et al., 2011). RCC2 promotes directional cell migration by binding to coronin-1C, which is crucial for RAC1 activation (Byron et al., 2011; Williamson et al., 2014).

RCC2 plays a role in α5β1-FN-signaling network and physically interacts and deactivates Rac1 (Yenjerla et al., 2013; Wu et al., 2018). Rac1, a small GTPase, interacts with RCC2's RCC1 like domain through its unique β-hairpin and participates in membrane protrusion (Wu et al., 2018). Moreover, RCC2 interacts with and deactivates Arf6, which is involved in integrin-dependent membrane trafficking (Pankov et al., 2005; Humphries et al., 2009; Song et al., 2018). Interaction between MENA (an actin regulator with roles in cell migration) and α5β1 integrin promotes cancer cell haptotaxis on fibronectin, leading to metastasis (Santiago-Medina and Yang, 2016). Therefore, α5β1 promotes progression of invasion of cancer cells. On the other hand, RCC2 plays a vital role in cellular response to changes in FN-concentrations (Song et al., 2018).

**Figure 4:**
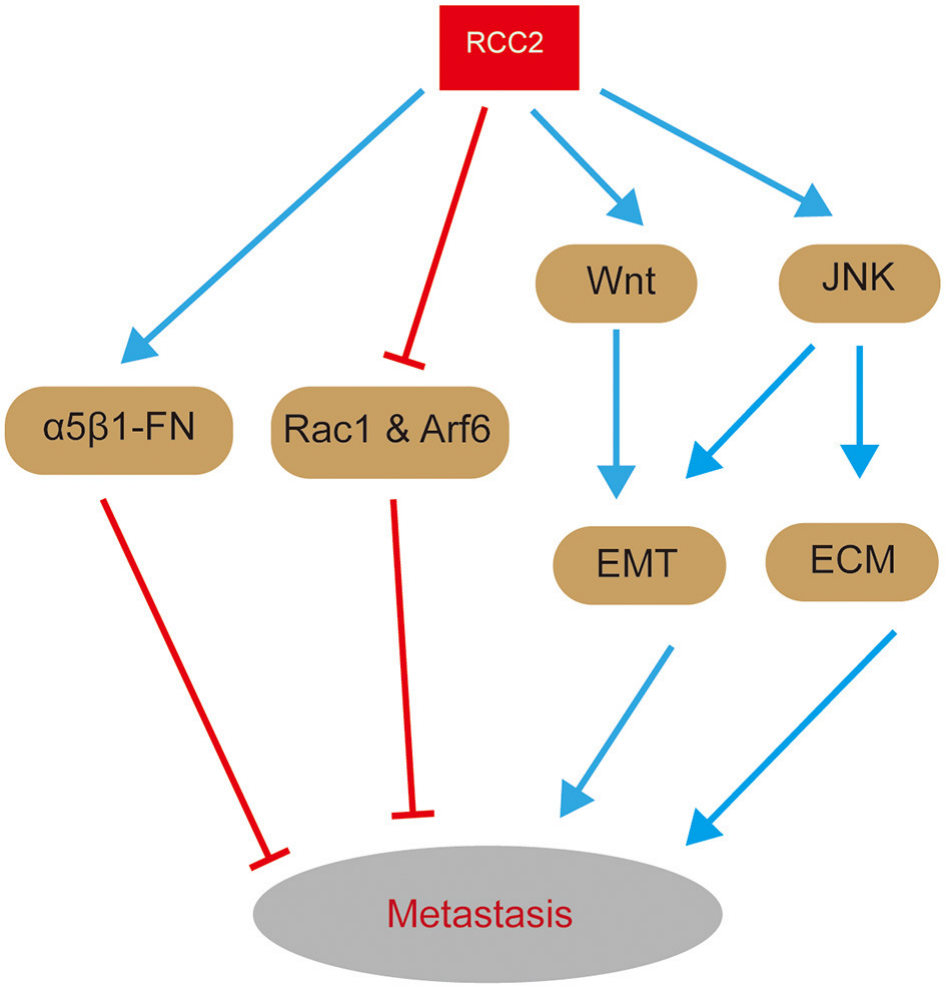
A graphical representation of the role of RCC2 in signaling pathways related with metastasis. On one hand, RCC2 inhibits activation of Rac1 and Arf6 and ensures proper function of α5β1-FN-signaling network, thus inhibiting metastasis. On the other hand, RCC2 promotes metastasis by regulating EMT and ECM remodeling through MAPK-JNK and Wnt-signaling pathways.

Expression of RCC2 is decreased by low levels of p53, which enhances activation of Rac1 and Arf6, and inhibition of cellular recognition of FN-concentrations (Pankov et al., 2005). This substrate surface ECM content concentrations leads to deterioration of haptotaxis. Activated Rac1 mediates random migration by promoting formation of peripheral lamellae, thus facilitating invasion of cancer cells (Pankov et al., 2005). Besides, haptotaxis functions as a sensor of varying concentration of underlying ECM, and inhibitory matrix cues cannot be sensed by deteriorated haptotaxis, resulting in random cell migration and tumor cell metastasis (Chan et al., 2014). Therefore, RCC2 inhibits tumor cell metastasis by inhibiting activation of small GTPases, Rac1, and Arf6, and by promoting α5β1-FN-signaling network and cellular recognition to FN concentrations (Figure 4).

Overexpression of RCC2 plays a functional role in LUAD metastasis. Overexpression of RCC2 increases expression of epithelial-mesenchymal transition (EMT) markers (N-cadherin and α-SMA), EMT-related transcription factors (Snail and Slug), and MMPs in LUAD (Pang et al., 2017). In addition, it decreases expression of β-catenin and E-cadherin, implying that RCC2 regulates EMT and ECM remodeling (Pang et al., 2017). Moreover, EMT and ECM remodeling are critical in promotion of tumor invasion and metastasis. RCC2-transfected cells increases expression of activated JNK, thus promoting migratory and invasive abilities of A549-RCC2 cells and RCC2-induced EMT and MMPs (Pang et al., 2017). These findings imply that MAPK-JNK signaling contributes to LUAD metastasis through induction by RCC2 (Figure 4). Therefore, overexpression of RCC2 promotes LUAD metastasis by inducing EMT and ECM remodeling through activation of MAPK-JNK signaling. Notably, mechanism of RCC2-induced metastasis in LUAD is similar to the mechanism in breast cancer. Although RCC2 promotes cell migration, by inducing EMT through activation of the Wnt-signaling pathway (Figure 4), it has been reported to promotes metastasis of ovarian cancer (Chen et al., 2019). Gulimire et al. report that direct targeting of RCC2 microRNA-331-3p (miR-331-3p) may function as a tumor suppressor in ovarian epithelial carcinoma (EOC) (Buranjiang et al., 2019). Therefore, RCC2 regulating tumor cell metastasis by activating a series of signaling pathways.

## RCC2 and DNA Damage

Few studies report on the relationship between RCC2 and DNA damage response (DDR). However, a previous study reports that RCC2 interacts with Ku86, which combines with Ku70 thus playing a core role in Non-homologous end joining (NHEJ), as detected by RP-RP MS, a precise mass spectrometric measurement (Zhou et al., 2010). Besides, another study reports that lncRNA LCPAT1 targets RCC2 during cigarette smoke extract (CSE) induced DNA damage (Gao et al., 2019). Interestingly, the study reported that a decrease in RCC2 expression decreases expression and foci numbers of γ-H2AX, a DNA damage marker, in CSE-treated Beas-2B cells, implying that loss of RCC2 exacerbates DNA damage (Gao et al., 2019). Association between RCC2 and γ-H2AX suggests that RCC2 is potentially associated with DDR. In summary, RCC2 may plays a role in DDR process, however further studies on the exact role of RCC2 in DDR should be carried out.

## Concluding Remarks

RCC2 plays key roles in regulation of cell cycle. Previous studies report that RCC2 is involved in all phases of the cell cycle. RCC2 plays a major role in the M phase compared with the interphase. In M phase, RCC2 associates with the chromosome and microtubule and functions as CPC or its member. Therefore, RCC2 promotes transition from prometaphase to metaphase by regulating the switch of inactive and active states of small G protein Rac1 and RalA. After transition, it becomes part of the telophase disc organelle for cell cleavage. Although studies report that low levels of RCC2 affect normal progression of interphase, the mechanism should be explored further.

RCC2 functions as an oncogene in numerous kinds of cancers by promoting progression of tumor cells, facilitates metastatic behaviors and induces therapeutic resistance in tumor cells. Expression level of RCC2 is significantly high in most common cancer types. The mechanism of RCC2 in CRC is complex. RCC2 knockdown in MSI group promotes tumor cell apoptosis. On the other hand, low expression level of RCC2 aggravates tumor cell metastasis through α5β1-FN-signaling pathway. In LUAD, RCC2 plays a significant role in MAPK-JNK signaling pathway, thus promoting LUAD cell proliferation and metastasis. RCC2 is a poor independent prognostic factor for LUAD patients. Expression level of RCC2 is associated with progression of EMT and regulation of activation of Wnt-signaling pathway, which promote development of breast cancer. In addition, RCC2 promotes cisplatin-resistance in ovarian cancer by regulating RalA signaling pathway. These findings show that RCC2 is an oncogene in tumorigenesis. Therefore, RCC2 is a potential therapeutic target for cancer treatment.

Furthermore, RCC2 has a potential role in modulating DNA damage process as it interacts with Ku86 and is associated withγ-H2AX. DDR is closely related to genome stability and tumorigenesis. However, the exact relationship between RCC2 and DNA damage should be explored further.

In summary, RCC2 plays an important role in cell cycle, cancer development, and therapeutic resistance of anticancer drugs. The role of RCC2 in the cell cycle is positively correlated with tumor formation and tumor cell sensitivity to therapies. Therefore, RCC2 is a potential biomarker for development of new and specific anti-cancer therapeutic strategies.

## Author Contributions

KG and CZ contributed to concept generation and drafting of the article. BL contributed to drafting of the article: HW and HZ contributed to drafting and approval of the article. FZ contributed to concept generation, drafting, and approval of the article. All authors read and approved the final manuscript.

## Conflict of Interest

The authors declare that the research was conducted in the absence of any commercial or financial relationships that could be construed as a potential conflict of interest.

## Abbreviations

RCC2: Regulator of Chromosome Condensation 2
CPC: Chromosomal passenger complex
GEF: Ran guanine exchange factor
LUAD: Lung adenocarcinoma
RLDs: RCC1-like domains
EMT: Epithelial-mesenchymal transition
MSS: Microsatellite stable
NLS: Nuclear localization signal
TD-60: Telophase Disc-60xl0^3^Mr
CRC: Colorectal cancer
MSI: Microsatellite instable
MMR: DNA mismatch repair
lncRNAs: Long non-coding RNAs
NSCLC: Non-small cell lung cancer
TCGA: The Cancer Genome Atlas
DNMT1: DNA methyltransferase 1
HCC: Hepatocellular carcinoma
XPO1: Exportin-1
DDP: Cisplatin
GBM: Glioblastoma
ECM: Extracellular matrix
MMPs: Matrix metalloproteinases
EOC: Epithelial ovarian carcinoma
DDR: DNA damage response
CSE: Cigarette smoke extract.
